# Inter-individual diversity and intra-individual stability of amphetamine-induced sensitization of frequency-modulated 50-kHz vocalization in Sprague–Dawley rats

**DOI:** 10.1007/s00213-012-2658-4

**Published:** 2012-02-22

**Authors:** Ewa Taracha, Adam Hamed, Paweł Krząścik, Małgorzata Lehner, Anna Skórzewska, Adam Płaźnik, Stanisław J. Chrapusta

**Affiliations:** 1 Department of Neurochemistry, Institute of Psychiatry and Neurology, 9 Sobieskiego St, 02-957 Warsaw, Poland; 2 Department of Experimental and Clinical Pharmacology, Medical University of Warsaw, 26/28 Krakowskie Przedmieście St, 00-927 Warsaw, Poland; 3 Department of Experimental Pharmacology, Mossakowski Medical Research Centre, Polish Academy of Sciences, 5 Pawińskiego St, 02-106 Warsaw, Poland

**Keywords:** Amphetamine, Drug dependence, Frequency-modulated ultrasonic vocalization, Inter-individual differences, Predictive power, Reward, Sensitization, Two-injection protocol of sensitization

## Abstract

**Rationale:**

Propensity for drug dependence shows great diversity that is related to intrinsic neurobiological factors. This diversity is important both for the understanding of these traits and for the development of therapies.

**Objectives:**

The goals of the study were (1) to define, using ultrasonic vocalization characteristics, inter-individual differences in rats’ propensity for sensitization to amphetamine, (2) to test whether possible resistance to this effect could be overcome with repetitive treatment, and (3) to seek useful predictors of the propensity.

**Methods:**

Rats were subject to tests meant to characterize their anxiety, pain sensitivity, and responses to novelty and natural rewards. Then they were subject to the so-called two-injection protocol of sensitization (using amphetamine) followed by 2 weeks of daily amphetamine treatment, 2-week withdrawal, and final amphetamine challenge. The development and outcome of sensitization were monitored by measuring 50-kHz vocalization.

**Results:**

The two-injection protocol yielded three patterns of changes in the frequency-modulated 50-kHz vocalization response to amphetamine. These patterns persisted after completion of the extended drug treatment. Rats with lower sensitivity to pain or with longer latency of their vocalization response to the first drug exposure showed an increased propensity for ultrasonic vocalization sensitization.

**Conclusion:**

Vulnerability to sensitization of frequency-modulated 50-kHz vocalization response of Sprague–Dawley rats to amphetamine, which supposedly reflects rats’ propensity for amphetamine dependence, shows large inter-individual diversity. Resistance to this effect, which is evident in a majority of the rats, cannot be overcome even with prolonged intermittent drug treatment under the conditions (novelty) that promote sensitization.

## Introduction

Of the large number of humans who experiment with addictive drugs, relatively few eventually become addicted (Zuckerman 1984; Le Moal and Koob 2007). This subpopulation also shows resistance to addiction therapy. The bases of these peculiarities are not clear, but it is generally recognized that the emergence of drug addiction is related, among others, to some pre-requisite, intrinsic biological factors. Such factors likely involve genetic background and/or a dysfunction in some neurotransmitter/neuromodulator system(s) (Bardo et al. 1996; Johansson and Hansen 2001; Cain et al. 2005; Sinha 2008; Taracha et al. 2011). Diversity of these factors and their combinations translates into heterogeneity with regard both to the propensity for addictions and to the sensitivity to the respective therapies. This heterogeneity is supported by both human and animal studies (Zuckerman 1984; Kalinichev et al. 2004; Kabbaj 2004, 2006; Cain et al. 2005; Pelloux et al. 2006; Everitt et al. 2008; Flagel et al. 2010).

The key attribute of addictive drugs is their ability for stimulating brain reward system(s) and for long-lasting potentiation (i.e., sensitization) of this effect. In laboratory rodents, these effects were usually assessed indirectly, e.g., using conditioned place preference or locomotor activity tests. Lately, the options for detecting and assessing affective states in rats and mice have been greatly enriched with methods based on measuring ultrasonic vocalization (USV) that is a common way of communication in these species (Knutson et al. 2002; Brudzynski 2007, 2009; Wang et al. 2008; Burgdorf et al. 2011). Adult rats vocalize in two frequency bands that are usually called 22 and 50 kHz, but actually range from 18 to 22 kHz and from ~30 to >100 kHz, respectively. The former (termed aversive or alarm) supposedly reflects negative affective states, e.g., fear or perception of danger from a predator. The higher frequency band calls, which can be highly modulated, usually associate with positive affective states evoked by naturally rewarding events (or anticipation of such), e.g., by access to food, social contacts with conspecifics, sex, or fresh bedding (Panksepp and Burgdorf 2000; Bialy et al. 2000; Brudzynski and Pniak 2002; Wang et al. 2008; Natusch and Schwarting 2010). They can also be induced by addictive drugs, e.g., cocaine (Maier et al. 2010; Barker et al. 2010; Ma et al. 2010) or amphetamine (Burgdorf et al. 2001b; Thompson et al. 2006; Wang et al. 2008; Wright et al. 2010, 2012; Brudzynski et al. 2011), or by drug context (Burgdorf et al. 2001a; Ma et al. 2010). Notably, 50-kHz USV requires activation of the mesolimbic dopamine system (Wintink and Brudzynski 2001; Thompson et al. 2006; Brudzynski 2009) that plays a key role in the rewarding effect of these drugs.

Similarly to certain non-vocalizational behavioral responses, appetitive USV can show sensitization after repeated exposure to psychoactive drugs (Barker et al. 2010; Ahrens et al. 2009). Moreover, like the propensity for addictions, 50-kHz vocalization response to non-pharmacological stimuli shows major inter-individual diversity, but little intra-individual variability (Schwarting et al. 2007; Mällo et al. 2007), implying a substantial role of genetic milieu (Brunelli and Hofer 2007; Burgdorf et al. 2009). These similarities may herald the utility of 50-kHz vocalization characteristics as an index of vulnerability to drug dependence.

This report presents an attempt to define individual differences in rats’ propensity for USV sensitization to amphetamine. We assumed that this sensitization might be achieved with the two-injection protocol of sensitization (TIPS) that has been successfully used for locomotor sensitization of mice to morphine and cocaine (Valjent et al. 2010). We were also curious whether possible resistance to this effect could be overcome with repetitive exposure, and we sought USV and perhaps other behavioral characteristics that would help predict USV sensitization to amphetamine. We hypothesized that there may be a link between USV sensitization to the drug and reactivity both to novelty, seeking of which is an established risk factor for drug abuse/dependence (Piazza et al. 1989; Kabbaj 2006), and to some naturally rewarding events, e.g., to post-isolation contact with a cage-mate (Panksepp and Burgdorf 2000; Brudzynski and Pniak 2002; Hamed et al. 2009). A vital role in controlling various effects of amphetamine and its active congeners plays the brain opioid system (Gianoulakis 2004; Burgdorf et al. 2009; Tien and Ho 2011), which is well-known to be responsible for reactivity to pain; for a review see Bodnar (2011). There is also considerable evidence showing a variety of anxiety-related actions of amphetamine (Dawson et al. 1995; Biala and Kruk 2007; Biala et al. 2009; Kitanaka et al. 2008; Barr et al. 2010; Ennaceur et al. 2010). Hence, we assumed that anxiety and reactivity to pain might be other likely predictors of USV sensitization to the drug.

## Materials and methods

### Animals

Twenty-two male Sprague–Dawley rats from the stock of the Polish Academy of Sciences Mossakowski Medical Research Centre, Warsaw, Poland were used for the study. The rats were 6–7 weeks old on arrival (initial body weight 142–174 g) and were housed six or four per opaque plastic cage (55 × 33 cm floor size) in a temperature- and humidity-controlled room (21 ± 2°C, 60–70% relative humidity) under a 12-h light/12-h dark cycle (lights on at 7 a.m.). They had ad libitum access to tap water and standard laboratory rodent chow and weighed 293–348 g (mean ± SD: 308 ± 13 g) at the beginning of drug treatment. Before the study, the rats were given ten “daily” (excepting weekends) sessions of handling and habituation to the testing milieu. The sessions included transferring the rats in their home cages to the testing room, placing them singly in a cage identical to the home cage, but with no bedding, and gently stroking them when on the experimenter’s hands and in the cage (for about 1 min each). The cage was cleaned and wiped with ethanol after each rat. During the first handling session, the rats were monitored for USV because we expected some aversive vocalization to be provoked by the encounter with unknown humans (Brudzynski 2009).

### Drugs

D-Amphetamine sulfate (Sigma-Aldrich) was dissolved (2 mg/ml) in sterile aqueous 0.9% NaCl solution and was injected to the rats intraperitoneally at the dose of 2 mg/kg.

### Experimental design

**Fig. 1:**
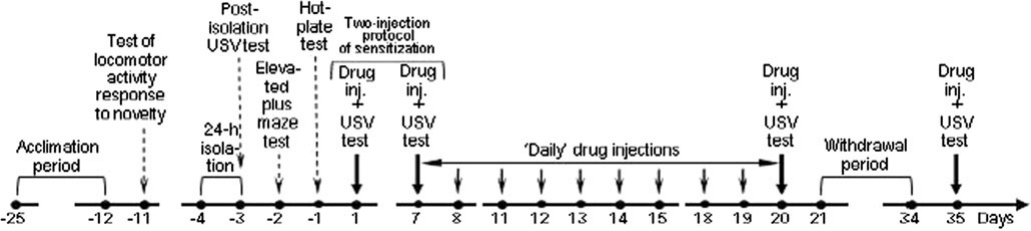
Scheme of experimental design

The scheme of the experimental design is shown in Fig. 1. The sequence of the tests preceding the beginning of amphetamine treatment (for tests’ details, see next sections) was arranged according to their estimated degree of invasiveness, beginning with the least invasive one, to minimize possible confounding effect on the next tests. After each of the tests, the rats were instantly returned to their home cages in the housing room. On the first day of drug treatment, they were brought to the testing room again, placed singly in clean clear plastic cages (56 × 34 cm floor size) with no bedding, tested for USV just prior to and after drug injection (for 10 and 20 min, respectively), and then returned to their home cages in the housing room for a 5-day break from injections and testing. Next, the rats were given the drug “daily” (except weekends, a total of ten doses) after transferring them (all cage-mates together, except on USV testing days) to clean cages (as above) in the testing room. One hour after each drug injection, the rats were returned to the housing room, except that after the first and the tenth dose, they were tested again for USV as above. During the 2-week withdrawal that followed the tenth daily dose, the rats stayed in the housing room. Then they were given the final amphetamine dose and tested for USV as usual. All the tests and injections were done during the light phase of the rats’ daily cycle. The study was designed and performed in accordance both with the European Union directive on the protection of laboratory animals (86/609 EEC) and with the current laws of Poland, and all animal use procedures were approved by the Bioethical Committee of the Medical University of Warsaw.

### Testing

#### Locomotor activity response to novelty (LA_nov_)

The rats were brought in their home cages to the testing room, placed singly in fresh opaque plastic cages (56 × 34 cm floor size) with no bedding, and immediately tested for locomotor activity (distance covered) for 15 min using a model YR-600 1/3 in. CCD 540 TVL camera (Sony, Japan) fixed to the room ceiling and a PC equipped with the EthoVision® XT Video Tracking System v. 7 (Noldus Information Technology B.V., Wageningen, The Netherlands).

#### Elevated plus maze (EPM) test

The EPM apparatus was made of wood and consisted of two opposed open arms and two opposed walled arms (arm floor sizes, 50 × 10 cm) and an open square (10 × 10 cm) in the center. The maze was elevated 50 cm above the room floor, was lit with a dim red light from a model Philips PF212E*1E bulb fixed 1.7 m above the floor of the EPM apparatus, and was monitored with the aforementioned video system. The rats were transferred singly to the testing room, placed on the central square, facing an open arm, and had 5 min access to the maze. Video recordings were then used to calculate the relative time spent on the open arms (expressed as percentage of total time spent in the apparatus).

#### Hot-plate (HP) test

To prevent rats associating the HP test-related, relatively strong stress with the regular testing room, the test was performed in a separate room, using a model HP-01 instrument (COTM, Białystok, Poland). Plate temperature was kept at 56 ± 0.1°C and the cut-off time was set at 30 s to prevent tissue damage. Rats were brought singly to the room and placed with all four paws on the plate, and the latency time to licking or shaking a fore or hind paw was measured.

#### USV recording

Excepting the USV_pi_ test (see below), USV calls were recorded using a single condenser microphone CM16/CMPA (Avisoft Bioacoustics, Berlin, Germany) placed face down on the wire cover of the cage. The microphone was sensitive to frequencies of 15–180 kHz, had a flat response characteristic (±6 dB) in the 25–140-kHz range, and was connected to a custom-made amplifier of the following characteristics: frequency response ±0.1 dB (0.3–100 kHz), input impedance 600 Ω, and voltage gain 16 V/V (12 dB). The amplified signal was sent to an adjacent (observer-occupied) room and passed through a custom-made anti-aliasing filter. The filtered signal was sent to a PC equipped with a model PCI-703-16A acquisition board (14-bit, 400 kHz; Eagle Technology, WI, USA) and custom-written software (Rat-Rec Pro 5.0), processed using a fast Fourier transform (1024 or 512, Hamming or Hann window) and displayed as a color spectrogram. Frequency-modulated (FM) 50-kHz calls (“trills”) and non-FM (“flat”) 50-kHz calls were identified using the characteristics given in earlier reports of the relevant rat studies (Wöhr et al. 2008; Ahrens et al. 2009; Wright et al. 2010). Each signal was manually marked to be included in the automated parameter measurement that included the following characteristics: number, mean and summary duration, frequency bandwidth and mean peak frequency of FM 50-kHz calls, and number of non-FM 50-kHz calls.

#### Post-isolation USV (USV_pi_) test

After a 24-h isolation (see the experimental design above), the rats were brought (in pairs, each rat in his isolation cage) to the testing room. The testing chamber consisted of the respective home cage (with “old” bedding in place) that was divided crosswise into two equal parts with a barrier composed of two pieces of 6-mm-thick plywood separated with a 3-cm-thick sheet of polyurethane foam. One of the plywood pieces had two horizontal rows of *ϕ* = 6-mm holes located 7.5 cm (eight holes) and 9.5 cm (six holes) from the lower edge; the holes were drilled 2 cm apart (center to center) and were located symmetrically in relation to the cage width. All three barrier parts closely fitted the cage cross-section, with the upper rim of each part protruding 20 cm above the cage rim and 10 cm sidewise on both sides. During the test, each cage section was covered with a separate wire cover. There was one ultrasound microphone placed on each cover, facing down. USV recording was performed for 10 min with all barrier parts in the “shut” position, then the solid plywood piece and the foam sheet were raised to level their lower edges with the cage rim, and the recording was continued for another 10 min. The perforated plywood piece, which remained in the “down” position to keep each rat in its assigned cage section, did not prevent the microphones from occasional collecting USV from the rat who stayed on the opposite side of the barrier; however, the “bias” calls were easily discerned by their low volume when comparing the respective “paired” sonograms.

### Statistics

Data are expressed as the mean ± SEM except when specified otherwise or shown individually. USV rate data (numbers of FM 50-kHz calls/time unit) were analyzed by a two-way or three-way ANOVA as needed, followed by post-hoc Tukey’s test when appropriate, except that “anticipatory” USV rate data, because of extreme deviation from normal distribution, were analyzed using nonparametric tests (Friedman’s ANOVA or Kruskal–Wallis ANOVA followed by the Dunn test of multiple comparisons when appropriate). For the analysis of predictive power of the various tests utilized, the rats were classified into low and high responders using “median split”. In all cases, a *p* < 0.05 was considered significant. All statistical analyses were performed using the Statistica v. 7.1 software package (StatSoft Inc., Tulsa, OK, USA).

## Results

Over the study period, rats showed no perturbation in their weight gaining and no other signs that might have suggested health problems. No 50-kHz call was detected during the first handling/habituation session, whereas there were four 20-kHz calls, all from a single rat.

Preliminary two-way repeated measures ANOVA of FM 50-kHz response to amphetamine (expressed as the number of calls per 2-min interval) for the entire rat cohort and all 4 days of USV testing yielded significant effects of post-drug session time (*F*_9, 189_ = 8.15, *p* < 0.001), day (*F*_3, 63_ = 3.24, *p* = 0.028), and day × post-drug session time interaction (*F*_27, 567_ = 4.99, *p* < 0.001).

### FM 50-kHz vocalization effects of TIPS

**Fig. 2:**
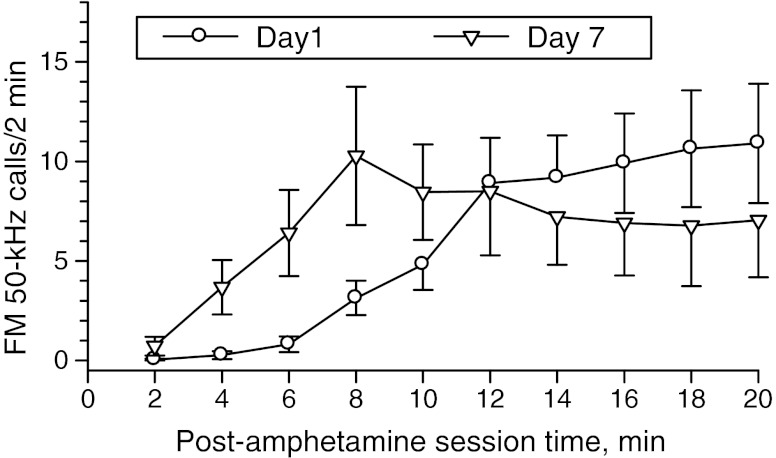
FM 50-kHz calling response to amphetamine two-injection protocol of sensitization in non-categorized study cohort. Two-way ANOVA—day effect: *F*_1, 21_ = 0.16, *p* = 0.70; post-drug session time effect: *F*_9, 189_ = 7.24, *p* < 0.001; day × post-drug session time interaction effect: *F*_9, 189_ = 3.20, *p* = 0.001

**Fig. 3:**
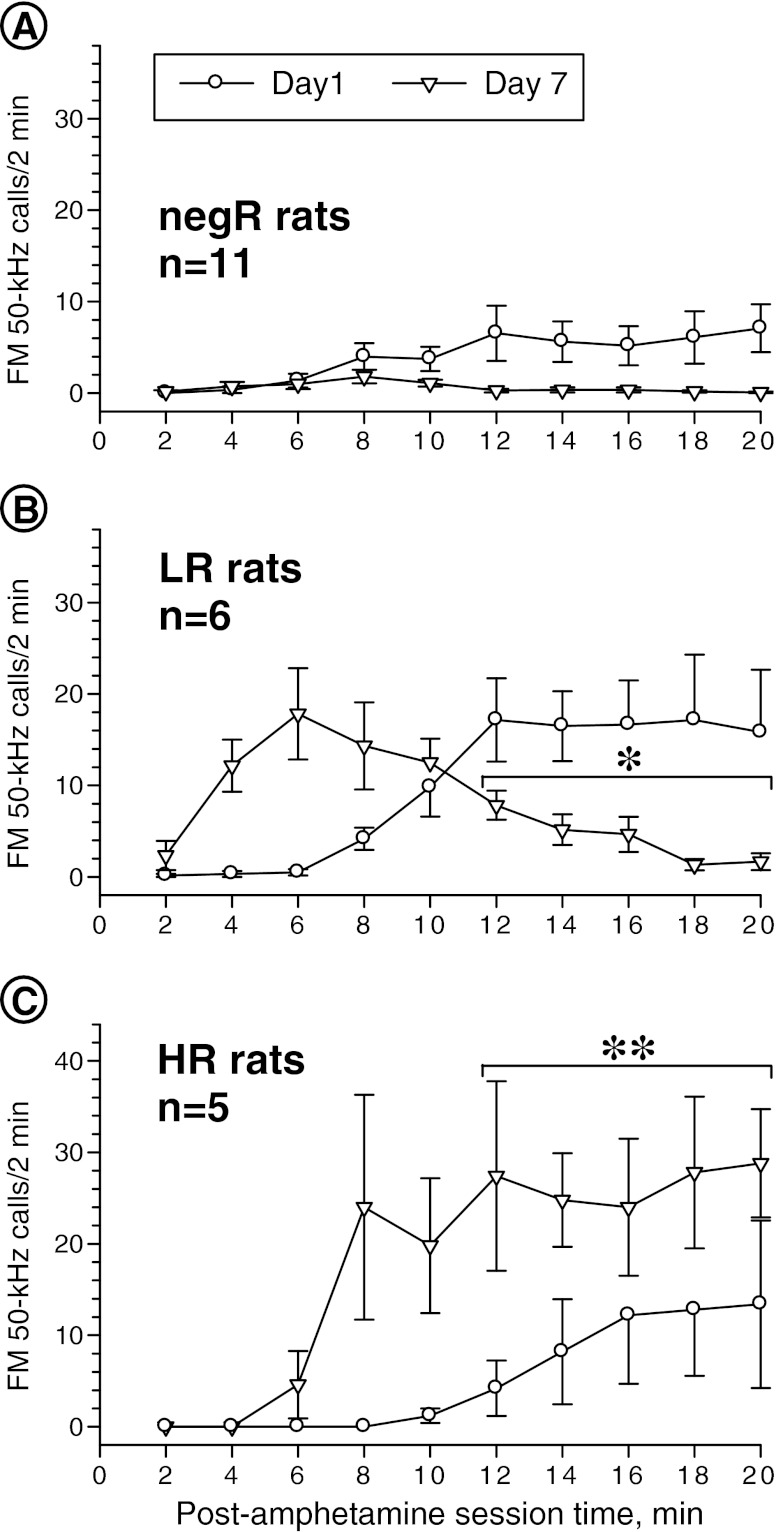
Patterns of FM 50-kHz USV responses to amphetamine two-injection protocol of sensitization. **a** Averaged response profiles of negative responders (*negR*); **b** averaged response profiles of low responders (*LR*); **c** averaged response profiles of high responders (*HR*). Please note that the averaged 2-min interval response profiles are shown only for illustration and the ANOVA results shown refer to 10-min time “blocks”. Three-way ANOVA—group effect: *F*_2, 19_ = 7.19, *p* = 0.005; day effect: *F*_1, 19_ = 4.16, *p* = 0.055; post-drug session time effect: *F*_1, 19_ = 15.23, *p* = 0.001; day × group interaction effect: *F*_2, 19_ = 14.22, *p* < 0.001; group × post-drug session time interaction effect: *F*_2, 19_ = 4.94, *p* = 0.019; day × post-drug session time interaction effect: *F*_2, 19_ = 9.04, *p* = 0.007; day × post-drug session time × group interaction effect: *F*_2, 19_ = 12.31, *p* < 0.001. *p < 0.05, **p < 0.01 vs. the corresponding 10-min block value for day 1; the Tukey test

The first amphetamine dose evoked a continuous increase in USV rate, with a tendency to plateau during the other half of the session, whereas the second dose (day 7) resulted in maximum USV rate at 8 min, followed by a slight decrease with a tendency to plateau at the end of the session (see Fig. 2). Repeated measures two-way ANOVA of the USV response data for the entire study cohort for days 1 and 7 showed no sensitization. However, the difference in the time course of the USV responses to the two drug doses, which was evidenced by statistically significant day × post-drug session time interaction effect, has prompted us to take a closer look at individual rat responses (not shown). Visual inspection revealed three patterns of changes. Half (*N* = 11) of the cohort studied showed a small increase in USV rate beginning at about 6 min and plateauing in the second half of the day 1 session. These rats showed poor response to the second dose, and their mean day 7 FM 50-kHz rate was below that found on day 1 (Fig. 3a); hence, they were termed “negative responders” (negR). Six rats showed a much larger increase in USV rate after the first drug dose, which lasted until the end of the session. These rats showed similar, but almost instant increase in USV rate after the second dose, but this response began to wane about 6 min post-drug and faded away before the session’s end (Fig. 3b); hence, they were termed “low responders” (LR). The remaining five rats showed moderate and much delayed increases in USV rate after the first drug dose, but their USV response to the next dose began noticeably earlier, was much stronger, and lasted to the end of the session with no sign of abating (Fig. 3c); we termed these rats “high responders” (HR).

### FM 50-kHz vocalization effects of repetitive amphetamine treatment

**Fig. 4:**
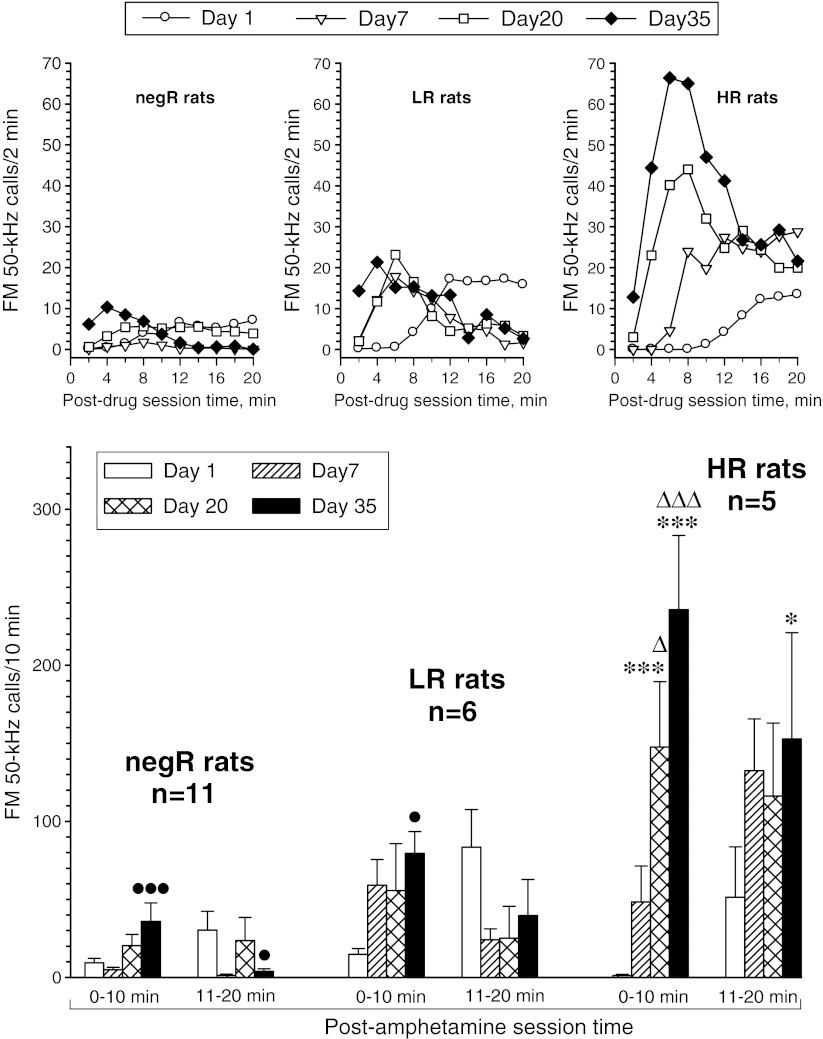
Effect of repetitive amphetamine treatment on FM 50-kHz USV response profiles in various rat subsets (*negR* negative responders, *LR* low responders, *HR* high responders). The *upper panels* are for illustration purpose only; hence, error bars are not shown. The ANOVA results given below refer to the data shown in the *lower panels*. Three-way ANOVA—group effect: *F*_2, 19_ = 14.88, *p* < 0.001; day effect: *F*_3, 57_ = 8.89, *p* < 0.001; post-drug session time effect: *F*_1, 19_ = 0.35, *p* = 0.56; day × group interaction effect: *F*_6, 57_ = 6.24, *p* < 0.001; group × post-drug session time effect: *F*_2, 19_ = 0.90, *p* = 0.42; day × post-drug session time interaction effect: *F*_3, 57_ = 11.90, *p* < 0.001; day × group × post-drug session time interaction effect: *F*_6, 57_ = 3.59, *p* = 0.004. *p < 0.05, ***p < 0.001 vs. the respective day 1 10-min block value; Δ*p* < 0.05, ΔΔΔ*p* < 0.001 vs. the respective day 7 10-min block value; •*p* < 0.05, •••*p* < 0.001 vs. the corresponding HR group value; the Tukey test

We attempted to analyze USV rate response data across all 4 days of testing for each rat subset separately by a repeated measures two-way ANOVA with treatment day and post-drug session time as repeated measures factors. The analysis using 2-min interval data was not feasible for the negR and HR subsets, as the software used reported no variability of the dependent variable; hence, the 2-min interval data were merged into two 10-min “blocks”. The respective two-way ANOVA re-analysis yielded significant effect of repetitive drug treatment on USV time course in all three subsets (day × post-drug session time interaction effect—negR rats: *F*_3, 30_ = 4.22, *p* = 0.013; LR rats: *F*_1, 15_ = 5.20, *p* = 0.012; HR rats: *F*_3, 12_ = 4.05, *p* = 0.033). The treatment transiently (day 7) suppressed the USV rate response in negR rats (day effect: *F*_3, 30_ = 3.22, *p* = 0.037), did not significantly affect it in LR rats (day effect: *F*_3, 15_ = 0.48, *p* = 0.70), and markedly enhanced it in HR rats (day effect: *F*_3, 12_ = 4.85, *p* = 0.020). Notably, USV rate response of the HR rats to the final drug dose (day 35) was significantly stronger than that to the second dose, while no similar change was found in the other rat subsets. For a detailed analysis of within- and between-subset effects, see Fig. 4.

**Fig. 5:**
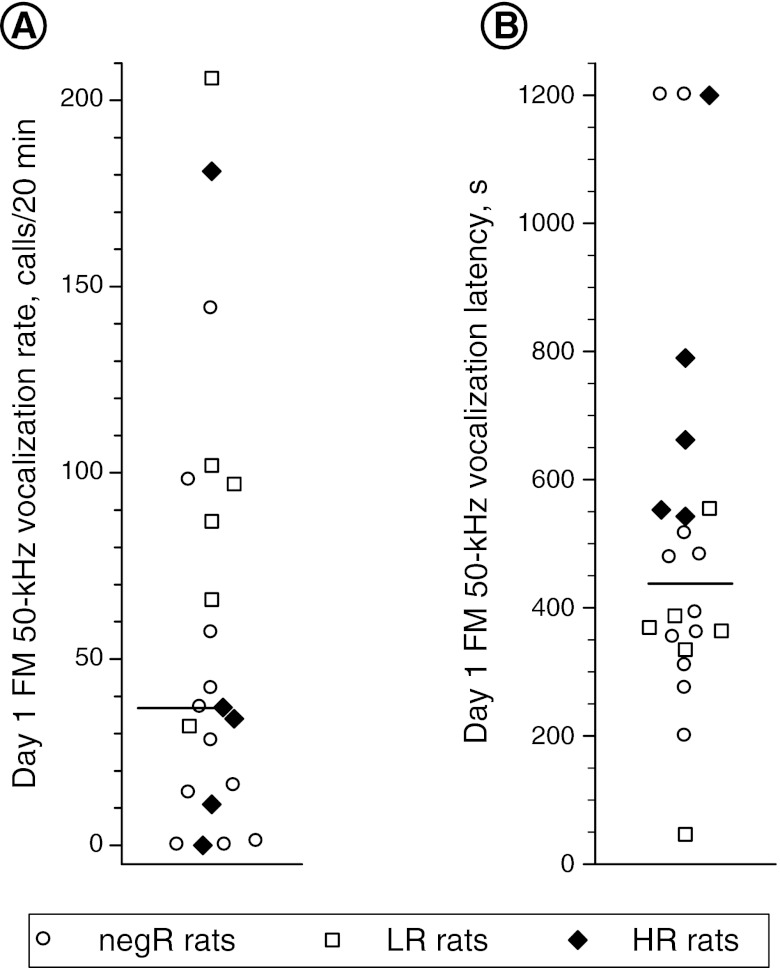
Median split categorization of the study cohort according to the rate of FM 50-kHz vocalization response to the first amphetamine dose (**a**) and the respective latency of the first post-amphetamine FM 50-kHz call (**b**)

Median split categorization of the study rats based on the rate of their FM 50-kHz calling response to the first drug dose (see Fig. 5a) yielded no predictive value with regard to sensitization of the FM 50-kHz response to amphetamine (three-way ANOVA—category effect: *F*_1, 20_ = 2.06, *p* = 0.17; category × day interaction effect: *F*_3, 60_ = 0.27, *p* = 0.85).

**Fig. 6:**
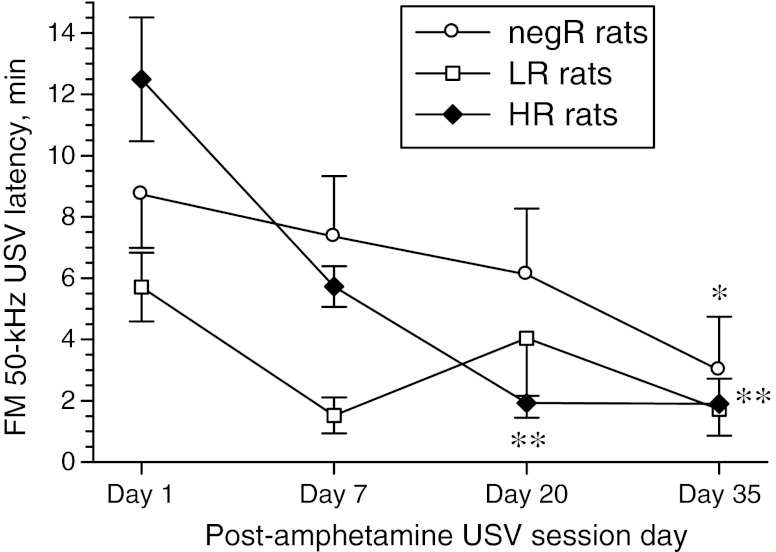
Effect of repetitive amphetamine treatment on the latency of the first post-drug FM 50-kHz call in various rat subsets identified based on their response to the TIPS protocol (*negR* negative responders, *LR* low responders, *HR* high responders). Two-way ANOVA—group effect: *F*_2, 19_ = 1.28, *p* = 0.30; day effect: *F*_3, 57_ = 11.31, *p* < 0.001; day × group interaction effect: *F*_6, 57_ = 2.14, *p* = 0.062. *p < 0.05, **p < 0.01 vs. the corresponding day 1 value; the Tukey test

**Fig. 7:**
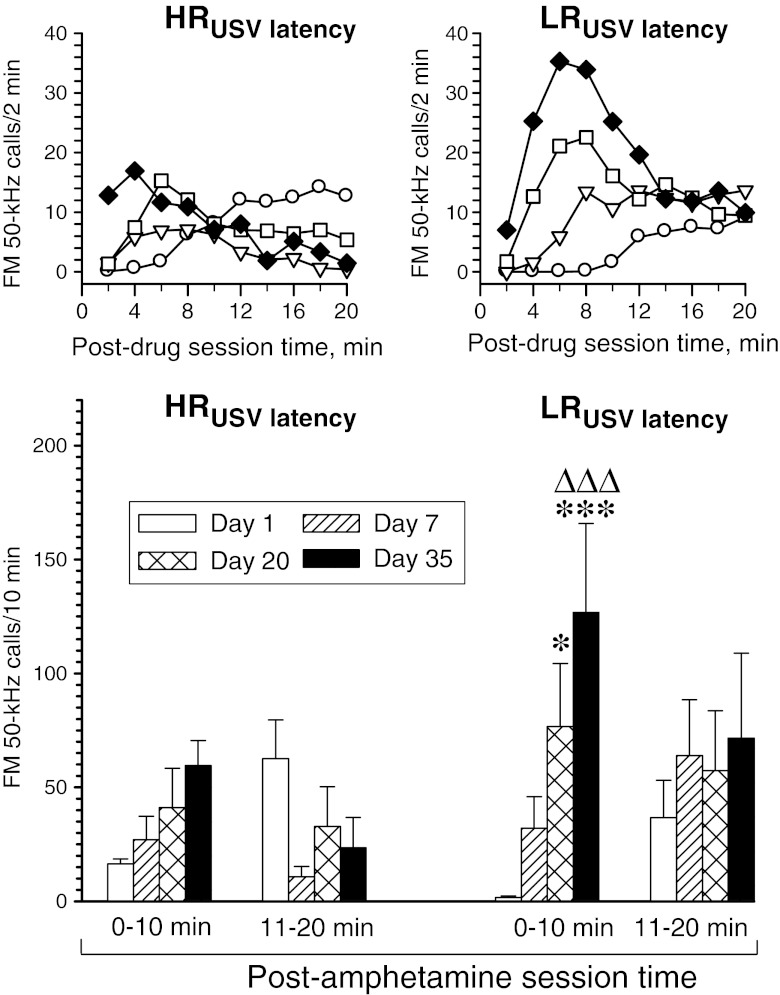
Effect of repetitive amphetamine treatment on FM 50-kHz USV rate response profiles in rats categorized by ranking in relation to the median latency time for the first post-drug USV recording session (*LR*_*USV latency*_ rats with above-median USV latency, *HR*_*USV latency*_ rats with below-median USV latency). The *upper panels* are for illustration purpose only; hence, for clarity, error bars are not shown. The ANOVA results given below refer to the data shown in the *lower panels*. Three-way ANOVA—category effect: *F*_1, 20_ = 1.36, *p* = 0.26; day effect: *F*_3, 60_ = 3.83, *p* = 0.014; post-drug session time effect: *F*_1, 20_ = 0.53, *p* = 0.48; day × category interaction effect: *F*_3, 60_ = 2.84, *p* = 0.045; category × post-drug session time interaction effect: *F*_1, 20_ = 0.05, *p* = 0.82; day × post-drug session time interaction effect: *F*_3, 60_ = 7.96, *p* < 0.001; day × post-drug session time × category interaction effect: *F*_3, 60_ = 1.47, *p* = 0.23. *p < 0.05, ***p < 0.001 vs. the corresponding day 1 10-min block value; ΔΔΔ*p* < 0.001 vs. the respective day 7 10-min block value; the Tukey test

Repetitive amphetamine treatment resulted in marked decreases in the latency time of the first post-drug FM 50-kHz call. This effect was most prominent in the HR rats (Fig. 6). Median split categorization of the study cohort by this latency (see Fig. 5b) showed that long latency was a significant predictor of USV sensitization (see Fig. 7).

### “Anticipatory” FM 50-kHz vocalization

**Fig. 8:**
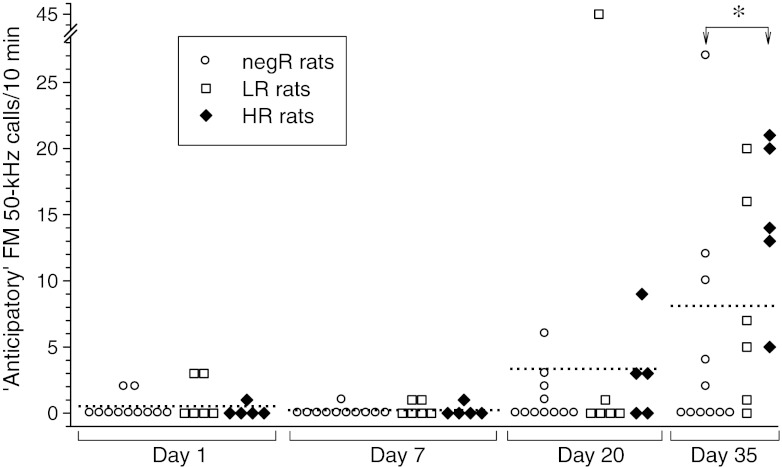
Effect of repetitive amphetamine treatment on FM 50-kHz USV rate during the 10-min sessions preceding drug injections. *Dotted lines* denote mean FM 50-kHz calling rates for the entire study cohort. *p < 0.05; Kruskal–Wallis ANOVA followed by the Dunn test

Very few FM 50-kHz calls were found during the sessions that preceded the first two amphetamine injections, but continued drug treatment resulted in markedly increased “anticipatory” FM 50-kHz calling (Fig. 8). This effect reached significance for the entire study cohort and the HR subset (Friedman’s ANOVA: *χ* ^2^_*df*=3, *N*=22_ = 22.1, *p* < 0.001 and *χ* ^2^_*df*=3, *N*=5_ = 11.8, *p* = 0.008, respectively). There was no significant between-group difference in the anticipatory USV rate (*p* ≥ 0.37, Kruskal–Wallis ANOVA) except before the last drug dose when the HR rats produced significantly more USVs than their negR counterparts (Fig. 8).

### FM 50-kHz vocalization characteristics

Summary duration of FM 50-kHz calls in individual rats (not shown) correlated highly (linear correlation coefficients from 0.98 to 1.00) with FM 50-kHz calling rates for 10-min recording session blocks on all testing days. Mean call duration for the first 10-min block ranged from 35.6 ± 2.5 ms (day 1) to 42.4 ± 1.1 ms (day 35) and that for the other 10-min block ranged between 34.8 ± 1.3 ms (day 1) and 42.4 ± 1.6 ms (day 7). Three-way ANOVA of mean call duration data, with rat subset (negR, LR, and HR) as the main factor, and testing day (days 1, 7, 20, and 35) and post-drug session time (two 10-min blocks) as the repeated measures factors yielded no significant effect of any factor (*p* ≥ 0.27) or factor interaction (*p* ≥ 0.30). Mean frequency bandwidth ranged from 12.6 ± 1.2 kHz (day 1) to 19.1 ± 1.0 kHz (day 35) for the first 10-min block and from 14.2 ± 0.9 kHz (day 1) to 17.8 ± 1.0 kHz (day 35) for the second block. Repeated measures three-way ANOVA of the frequency bandwidth data also showed no significant effect of individual factors (*p* ≥ 0.12) or of factor interactions (*p* ≥ 0.52). Mean call frequency ranged from 64.0 ± 1.9 kHz (day 1) to 65.2 ± 1.4 kHz (day 7) for the first 10-min block and from 66.2 ± 1.4 kHz (day 1) to 68.9 ± 1.6 kHz (day 7) for the other half of the sessions. The respective three-way ANOVA of mean FM 50-kHz call frequency data showed no significant effect of any factor (*p* ≥ 0.13) or factor interaction (*p* ≥ 0.19) as well.

### Rat categorization by USV_pi_ and non-USV testing

**Fig. 9:**
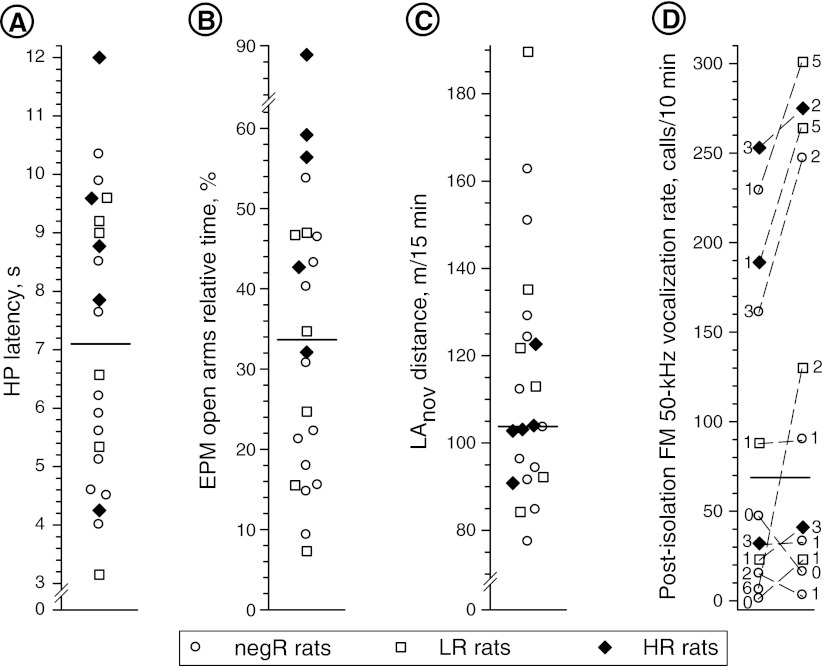
Median split categorization of the study cohort according to HP response latency (**a**), EPM open arms relative time (**b**), locomotor response to novel environment (**c**), and post-isolation encounter FM USV rate (**d**). Please note that the *dashed lines* in **d** denote individual rat pairs, whereas the *single-digit numbers at the symbols* denoting individual rats show the number of non-FM 50-kHz calls uttered by the respective rats during the encounter session

**Fig. 10:**
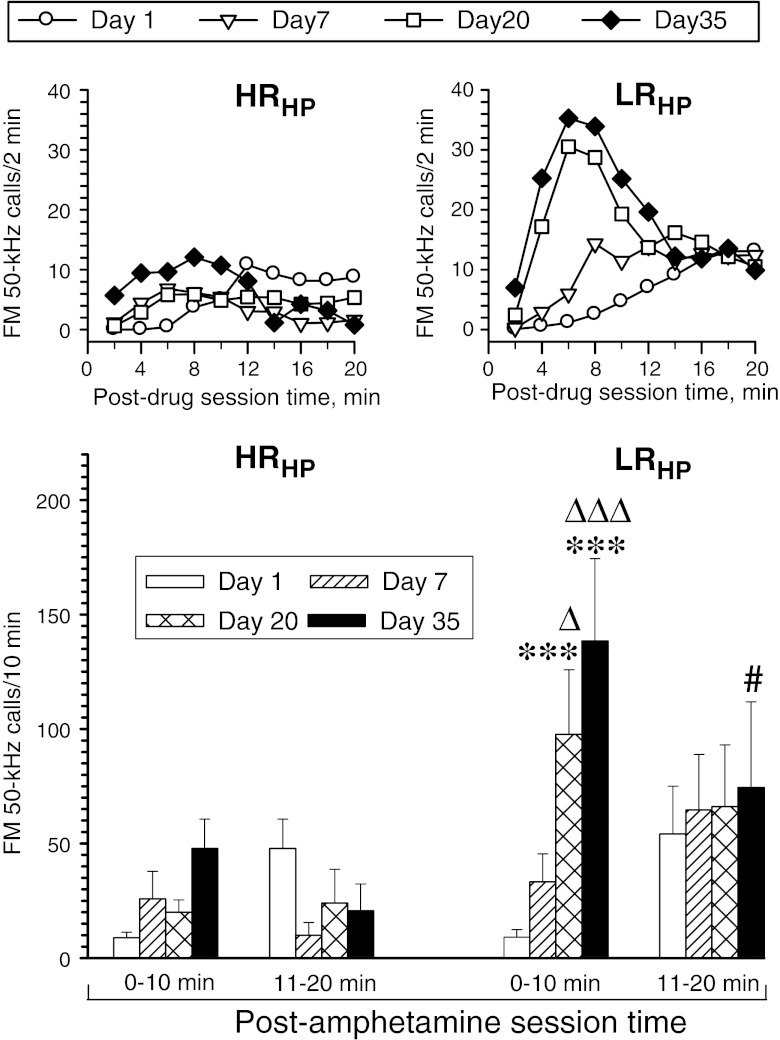
Effect of repetitive amphetamine treatment on FM 50-kHz USV rate response profiles in rat subsets defined by ranking in relation to the median HP response latency (*LR*_*HP*_ rats with above-median HP response latency, *HR*_*HP*_ rats with below-median HP response latency). The *upper panels* are for illustration purpose only; hence, for clarity, error bars are not shown. The ANOVA results given below refer to the data shown in the *lower panels*. Three-way ANOVA—day effect: *F*_3, 60_ = 3.76, *p* = 0.015; category effect: *F*_1, 20_ = 4.76, *p* = 0.041; post-drug session time effect: *F*_1, 20_ = 0.53, *p* = 0.47; day × category interaction effect: *F*_3, 60_ = 2.44, *p* = 0.073; category × post-drug session time interaction effect: *F*_1, 20_ = 0.30, *p* = 0.59; day × post-drug session time interaction effect: *F*_3, 60_ = 8.37, *p* < 0.001; day × post-drug session time × category interaction effect: *F*_3, 60_ = 2.57, *p* = 0.063. ***p < 0.001 vs. the corresponding day 1 10-min block value; Δ*p* < 0.05, ΔΔΔ*p* < 0.001 vs. the corresponding day 7 10-min block value; #*p* < 0.05 vs. the corresponding 0–10-min block value; the Tukey test

**Fig. 11:**
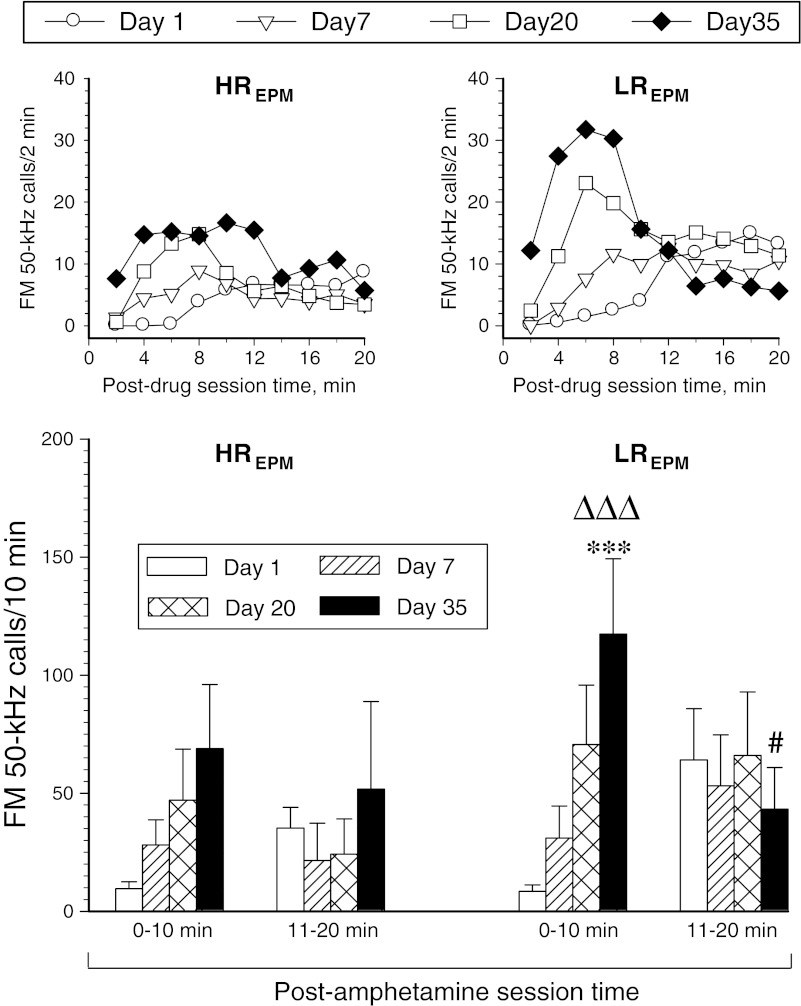
Effect of repetitive amphetamine treatment on FM 50-kHz USV rate response profiles in rat subsets categorized by rat ranking in relation to the median value of the EPM test (*HR*_*EPM*_ rats with above-median relative time spent on the open arms of the EPM, *LR*_*EPM*_ rats with below-median relative time spent on the open arms of the EPM). The *upper panels* are for illustration purpose only; hence, error bars are not shown. The ANOVA results given below refer to the data shown in the *lower panels*. Three-way ANOVA—category effect: *F*_1, 20_ = 1.01, *p* = 0.33; day effect: *F*_3, 60_ = 3.38, *p* = 0.024; post-drug session time effect: *F*_1, 20_ = 0.54, *p* = 0.47; day × category interaction effect: *F*_3, 60_ = 0.16, *p* = 0.92; day × post-drug session time interaction effect: *F*_3, 60_ = 8.45, *p* < 0.001; category × post-drug session time interaction effect: *F*_1, 20_ = 0.45, *p* = 0.51; day × post-drug session time × category interaction effect: *F*_3, 60_ = 2.78, *p* = 0.049. ***p < 0.001 vs. the corresponding day 1 10-min block value, ΔΔΔ*p* < 0.001 vs. the corresponding day 7 10-min block value; "#*p*<0.05 vs. the corresponding 0-10-min block value; the Tukey test

Median split categorization of the study rats into HP high responders and low responders (with below-median and above-median HP latency, respectively; Fig. 9a) showed significant predictive value for the sensitization of the FM 50-kHz response to amphetamine (see Fig. 10). Analogous categorization of this cohort into EPM high and low responders (low-anxiety and high-anxiety rats, respectively; Fig. 9b) yielded no significant prognostic value, but there was a significant increase in the rate of FM 50-kHz calling response to amphetamine in the EPM high responders at the end of drug treatment (see Fig. 11).

Median split categorizations based on results of the LA_nov_ (Fig. 9c) and USV_pi_ test (Fig. 9d) also showed no predictive value: LA_nov_ three-way ANOVA—category effect: *F*_1, 19_ = 0.03, *p* = 0.86; category × day interaction effect: *F*_3, 57_ = 0.43, *p* = 0.73; FM 50-kHz USV_pi_ rate three-way ANOVA—category effect: *F*_1, 20_ = 0.18, *p* = 0.67; category × day interaction effect: *F*_3, 60_ = 0.69, *p* = 0.56. A closer scrutiny of the FM 50-kHz rate has shown that it was strongly biased by an interaction between the test-paired rats. Despite large between-pair variability, seven of the tested pairs showed a good match in the number of FM 50-kHz calls emitted by the test-paired rats (rat A/rat B calls ratio—1.02–1.76, “B” being the rat who vocalized less); a major disparity was found for two pairs (calls ratios—21.7 and 23.0), and the other two pairs showed an intermediate discordance (calls ratios: 2.9 and 5.0). The rate of non-FM 50-kHz calling during the USV_pi_ test was negligibly small compared to that of FM 50-kHz calling, and there was no apparent relationship between these rates for any rat subset identified (see Fig. 9d).

## Discussion

Repeated administration of psychoactive drugs, especially in a novel environment (Badiani and Robinson 2004; Paolone et al. 2007), is well known to induce behavioral sensitization in laboratory rodents. In rats, this effect can be detected by testing either the locomotor activity (Robinson and Berridge 1993; Vezina and Queen 2000; Vanderschuren and Kalivas 2000; Taracha et al. 2008; Ahrens et al. 2009) or FM 50-kHz vocalization (Ahrens et al. 2009). However, the relationship between these measures is not yet clear; see Ahrens et al. (2009) and Mu et al. (2009).

Sensitization is usually assessed after a few weeks withdrawal from repeated psychoactive drug treatment. Yet even single doses of such drugs can initiate locomotor sensitization (Robinson et al. 1982; Jackson and Nutt 2001; Vanderschuren et al. 2001), especially when they are given in a unique, drug-specific context (Valjent et al. 2010). Using the TIPS protocol in mice, this effect was found to emerge within just 7 days following the priming cocaine dose and somewhat later after morphine treatment of mice (Valjent et al. 2010). We have used a similar protocol here, but the second amphetamine dose meant to reveal FM 50-kHz USV response sensitization was also the beginning of daily treatment intended both to overcome a possible resistance to the sensitization and to allow us comparing the effects of single and multiple exposures to the drug.

Notably, the TIPS protocol revealed no USV sensitization to amphetamine in unselected Sprague–Dawley rats. However, based on the data from individual USV recording sessions associated with this protocol, we have identified three rat subsets (negR, LR, and HR) that greatly differed in their propensity for USV-defined sensitization. During the period covered by the TIPS protocol, these subsets showed distinct changes in their respective patterns of FM 50-kHz calling response to amphetamine. The patterns established during this time showed no qualitative change over the course of further drug treatment. True sensitization of the USV response in the TIPS protocol emerged only in the HR rats. Notably, only these rats showed clear enhancement of this effect with continued treatment and withdrawal. In contrast, the remaining over three fourths of the study rats showed no sensitization and only minor changes in the characteristics of their responses to amphetamine during the continued treatment, revealing an amazing resistance to multiple amphetamine exposures. These results are in line with the evidence that a single dose of a psychoactive drug can be sufficient for the induction and maturation of the neurobiological changes needed for the expression of sensitized locomotor phenotype. They are also compatible with the view that potentiation of sensitized phenotype by continued drug exposure may result from either a progress in these processes, or from other phenomena (Valjent et al. 2010).

There were major changes in the rate of FM 50-kHz calling over the courses of all recording sessions. Similar changes after acute amphetamine treatment have been found in another study employing similarly long sessions (Wright et al. 2010), but could not be seen in the only other rat USV study on repeated amphetamine treatment (Ahrens et al. 2009) because of the short session time employed (5 min). It should be stressed that using these short sessions, at least some of our HR rats would have been categorized as low responders, whereas our LR rats would appear roughly equivalent to the rats that were reported to sensitize to the drug in the study of Ahrens et al. and would have been deemed “high responders”.

It is recognized that the rate of USV responses to different stimuli is related to some heritable factors and shows large individual variability (Brunelli 2005; Mällo et al. 2007; Burgdorf et al. 2009). One may ask if the differences we observed were really related to the reaction to amphetamine or rather to individual rats’ ability for USV. Our data suggest that rats’ propensity for USV sensitization to the drug is not related to the rate of their response to the stimuli that induce appetitive USV, e.g., to post-isolation contact with a cage-mate, but is defined by some neurobiological peculiarities that underlie their sensitivity to amphetamine.

Humans who are pleased with the acute effects of the first drug dose are supposedly more prone for voluntary intake of the next doses and thus show a higher risk for developing addictions (de Wit 1998; Gabbay et al. 2010). Alternatively, the inclination to drug addiction may be related to a deficit in the function of brain reward system(s) (Vetulani 2001; Winstanley et al. 2010; Koob 2012). Such deficits may render natural rewards insufficiently gratifying and generate the urge for more effective agents, including substances capable of “compensating” the deficits by acutely modifying brain chemistry. Hence, it was interesting to see whether rats’ vulnerability to USV sensitization is related to their sensitivity to pharmacological and natural rewards. Categorization based on the results of post-isolation USV test has failed in this respect. This outcome was likely to be blamed on pre-existing relationships between the test-paired rats, as it has been shown by others that appetitive USV from a rat can modify this behavior in conspecifics (Sadananda et al. 2008; Wöhr and Schwarting 2009).

The positive affective state (as evidenced by the emission of appetitive USV) elicited by the first drug dose also did not differentiate our rats with regard to their vulnerability to USV sensitization. Yet the long latency time for the first post-amphetamine FM 50-kHz call appeared a significant predictor of drug-induced USV sensitization. It is not possible to decisively state whether the latency is related to the perception of amphetamine as pleasant. However, the lack of predictive power of the categorizations based on the rate of USV response to the first drug dose and to the post-isolation contact with cage-mate suggests no link between amphetamine-induced USV sensitization and sensitivity to rewards.

FM 50-kHz vocalization is also assumed useful for evaluation of the affective states associated with anticipation of the next doses of addictive drugs (Ma et al. 2010); we have also found increased context-related USV rate in Sprague–Dawley rats repeatedly treated with morphine (Hamed et al., unpublished data from this laboratory). This postulation has been confirmed by statistical significance of the increases in anticipatory USV found after multi-dose treatment in both the HR subset and the entire rat cohort in our study. The remaining USV characteristics that have been examined in this study (mean call duration, summary calls duration, call frequency bandwidth, and mean peak frequency) showed remarkable stability. This finding is in agreement with the report of Wright et al. (2010). Notably, these USV features did not also differ between the various rat subsets identified.

Our results regarding sensitization of FM 50-kHz vocalization response to amphetamine are in general agreement with the report of Ahrens et al. (2009) despite much lower vocalization rate of our rats. Notably, our rats uttered also almost no 22-kHz (i.e., aversive) calls and no 50-kHz calls during the first handling/habituation session. This low calling rate, which also appertains to the FM 50-kHz vocalization that preceded the administration of consecutive drug doses (“anticipatory” USV), cannot be explained by different drug administration routes because there is a similar discrepancy between our data and those obtained in studies on acute USV effects of amphetamine (Wright et al. 2010, 2012). The reason of the disparity could be the fact that unlike most researchers, we carried out our experiments during the light phase of the rats’ daily cycle, i.e., during the period of low activity; this condition seems to be of great importance for behavioral effects of addictive drugs (see also Taracha et al. 2011). This supposition is supported by the fact that we have observed much more intense FM 50-kHz vocalization in Sprague–Dawley rats placed under dim red light during the light phase of their daily cycle, either with no additional treatment or after i.p. physiological saline injections (Hamed et al., unpublished data from this laboratory). Another cause of the said differences might be the different rat strain used. However, the relatively low rate of the “anticipatory” USV in our rats could be related also to a stronger conditioning effect in the other models (Maier et al. 2010; Ma et al. 2010).

The only non-USV-based characteristic that showed some promise as a predictor of the propensity for USV sensitization to amphetamine in this study was lower sensitivity to pain (as assessed by the HP test). This finding may be related to the fact that the brain opioid system, which plays a key role in reaction to pain, is also involved in controlling USV (Tien and Ho 2011) and some positive affective state-related effects of amphetamine (Wang and McGinty 1995; Olive et al. 2001; Wiskerke et al. 2011). However, the significance of the HP latency-based categorization of the study rats did not translate into a significant between-group difference in post-drug FM 50-kHz calling rate on any testing day (see Fig. 7). Hence, the prognostic power of HP test asks for verification, preferably in a larger cohort. Lack of prognostic value of the categorization based on the results of EPM testing may look somewhat surprising, as there is evidence suggesting that a lowered anxiety in this test is related to the propensity for cocaine dependence in rats (Bush and Vaccarino 2007). Lack of predictive power of the locomotor activity response to a novel environment in our study is more understandable, as the results of this test are highly dependent on the exact way it is performed and show major divergence between different studies (see Kabbaj 2006; Beckmann et al. 2011).

One might argue that the battery of pre-drug treatments applied to the rats in this study could have significantly biased the results of amphetamine treatment. However, since we took measures to minimize the confounding potential of these manipulations, and since all study rats were subjected to identical sequence of experimental manipulations, we believe that the non-pharmacological procedures had no considerable effect on the identified USV sensitization-related differences.

### Concluding remarks

We have found that the two-injection protocol of sensitization does allow to reliably define rat’s propensity for USV sensitization to amphetamine, provided the sensitization is assessed with consideration of both the changes in FM 50-kHz calling rate and the changes in the latency and duration of the USV response. USV recording sessions used for the evaluation should be of at least 20-min duration to avoid misjudgment. We have also shown that rats’ pain sensitivity and the latency of their FM 50-kHz vocalization response to the first drug dose have some prognostic power in relation to USV sensitization, at least for that to amphetamine. The most important results of this study are as follows: (1) the propensity of male Sprague–Dawley rats for FM 50-kHz USV sensitization to amphetamine is highly diversified, (2) rats showing this vulnerability account for but a minor subset of unselected males, and (3) the resistance of the remaining male Sprague–Dawley rats to this sensitization cannot be overcome even with repetitive drug treatment under the conditions that promote sensitization. These findings ask for a reassessment in a larger cohort, possibly using more detailed FM 50-kHz characteristics and longer USV recording sessions, as the relatively long-lasting high post-amphetamine FM 50-kHz vocalization rate in sensitized rats suggests that such approach might reveal additional characteristics of their response. It would also be interesting to see whether the timing of amphetamine treatment in relation to the rats’ daily cycle has a major impact on USV sensitization.
